# Colonization dynamics of subgingival microbiota in recently installed dental implants compared to healthy teeth in the same individual: a 6-month prospective observational study ^*^

**DOI:** 10.1590/1678-7757-2023-0134

**Published:** 2023-09-15

**Authors:** Carina Maciel SILVA-BOGHOSSIAN, Pablo Torquilho DUARTE, Denise Gome da SILVA, Talita Gomes Baêta LOURENÇO, Ana Paula Vieira COLOMBO

**Affiliations:** 1 Universidade Federal Rio de Janeiro Faculdade de Odontologia Departamento de Clínica Odontológica Rio de Janeiro Brasil Universidade Federal Rio de Janeiro, Faculdade de Odontologia, Departamento de Clínica Odontológica, Rio de Janeiro, Brasil.; 2 Universidade do Grande Rio Programa de Pós-graduação em Odontologia Duque de Caxias Rio de Janeiro Brasil Universidade do Grande Rio, Programa de Pós-graduação em Odontologia, Duque de Caxias, Rio de Janeiro, Brasil.; 3 Universidade Federal do Rio de Janeiro Instituto de Microbiologia Paulo de Góes Departamento de Microbiologia Médica Rio de Janeiro Brasil Universidade Federal do Rio de Janeiro, Instituto de Microbiologia Paulo de Góes, Departamento de Microbiologia Médica, Rio de Janeiro, Brasil.

**Keywords:** Dental implants, Microbiota, Molecular diagnostic techniques, DNA probes

## Abstract

**Objectives:**

To evaluate the colonization dynamics of subgingival microbiota established over six months around newly installed dental implants in periodontally healthy individuals, compared with their corresponding teeth.

**Methodology:**

Seventeen healthy individuals assigned to receive single dental implants participated in the study. Subgingival biofilm was sampled from all implant sites and contralateral/ antagonist teeth on days 7, 30, 90, and 180 after implant installation. Microbiological analysis was performed using the Checkerboard DNA-DNA hybridization technique for detection of classical oral taxa and non-oral microorganisms. Significant differences were estimated by Mann-Whitney and Friedman tests, while associations between implants/teeth and target species levels were assessed by linear regression analysis (LRA). Significance level was set at 5%.

**Results:**

Levels of some species were significantly higher in teeth compared to implants, respectively, at day 7 ( *V.parvula* , $6\times 10^{5}$ vs 3×105 ; *Milleri streptococci* , $2\times 10^{6}$ vs $6\times 10^{5}$ ; *Capnocytophaga* spp., $2\times 10^{6}$ vs $9\times 10^{5}$ ; *E.corrodens* , $2\times 10^{6}$ vs $5\times 10^{5}$ ; *N. mucosa* , $2\times 10^{6}$ vs $5\times 10^{5}$ ; *S.noxia* , $2\times 10^{6}$ vs $3\times 10^{5}$ ; *T.socranskii* , $2\times 10^{6}$ vs $5\times 10^{5}$ ; *H.alvei* , $4\times 10^{5}$ vs $2\times 10^{5}$ ; and *Neisseria* spp., $6\times 10^{5}$ vs $4\times 10^{4}$ ), day 30 ( *V.parvula* , $5\times 10^{5}$ vs 10 ^5^ ; *Capnocytophaga* spp., $1.3\times 10^{6}$ vs $6.8\times 10^{4}$ ; *F.periodonticum* , $2\times 10^{6}$ vs 10 ^6^ ; *S.noxia* , $6\times 10^{5}$ vs $2\times 10^{5}$ ; *H.alvei* , $8\times 10^{5}$ vs $9\times 10^{4}$ ; and *Neisseria* spp., $2\times 10^{5}$ vs 10 ^6^ ), day 120 ( *V.parvula* , $8\times 10^{5}$ vs $3\times 10^{5}$ ; *S.noxia* , $2\times 10^{6}$ vs 0; and *T.socranskii* , $3\times 10^{5}$ vs $8\times 10^{4}$ ), and day 180 ( *S.enterica* subsp. *enterica* serovar Typhi, $8\times 10^{6}$ vs $2\times 10^{6}$ ) (p<0.05). Implants showed significant increases over time in the levels of *F.nucleatum* , *Gemella* spp., *H.pylori* , *P.micra* , *S.aureus* , *S.liquefaciens* , and *T.forsythia* (p<0.05). LRA found that dental implants were negatively correlated with high levels of *S. noxia* and *V. parvula* (β=-0.5 to -0.3; p<0.05).

**Conclusions:**

Early submucosal microbiota is diverse and only a few species differ between teeth and implants in the same individual. Only 7 days after implant installation, a rich microbiota can be found in the peri-implant site. After six months of evaluation, teeth and implants show similar prevalence and levels of the target species, including known and new periodontopathic species.

## Introduction

The increasing number of individuals living with dental implants has brought clinical challenges for patients and professionals alike as eventually most patients may develop peri-implant mucositis or peri-implantitis, depending on several local and systemic factors related to the individual’s susceptibility.^1 , 2^ Peri-implant infections have as a primary etiological factor the overgrowth and persistence of pathogenic subgingival biofilm.^1 , 3 , 4^ Peri-implant microbial colonization occurs soon after implant installation,^5^ whereas late implant failures are related to a dysbiotic bacterial biofilm.^6 , 7^ Since the severity of peri-implantitis is associated with the established bacterial dysbiosis,^8^ maintaining balance between a bacterial biofilm and the host around dental implants is important to preserve peri-implant health.^9^ This biofilm-host balance becomes increasingly relevant in patients with a history of periodontitis, since periodontal pockets are a reservoir for periodontal pathogens, and their translocation to submucosal sites is more likely to occur.^4 , 10 - 13^

Previous studies have described the bacterial profile in early colonization around dental implants by DNA probes^5 , 14^ or standard culture techniques^15^ targeting usual periodontal pathogens, showing that red and orange complexes species have a similar detection frequency around implants and teeth with comparable probing depths.^5^ Overall, bacterial counts increase in the submucosal site a few weeks after implant installation.^14 , 16^ Comparisons between periodontitis and peri-implantitis have also found similarities in the subgingival and submucosal colonization by putative periodontal pathogens.^17 , 18^ Conversely, the microbial composition of healthy implants seems to differ significantly from peri-implantitis cases.^19^

Advances in culture-independent techniques for studying the subgingival microbiota have indicated novel microbial taxa that may play a role in periodontal or peri-implant disease pathogenesis, such as *Dialister* and *Filifactor.* ^16 - 18 , 20 , 21^ Importantly, most of the genomic sequencing data related to subgingival biofilm around implants are presented at the phyla and genera levels and not at the species level.^16 , 21 , 22^ A systematic review showed that available evidence in the literature is insufficient to provide a final answer concerning the microorganisms colonizing dental implants.^23^ Moreover, there is limited information on the colonization dynamics over the first months after implant installation regarding candidate periodontal pathogens and other bacteria of medical importance. Understanding the first stages of bacterial colonization for those new periodontal pathogens might be essential for developing effective strategies to prevent peri-implant diseases. Hence, we hypothesized that novel periodontal pathogens can colonize the submucosa around dental implants at early stages after installation. This study evaluated the kinetics of the submucosal microbiota colonization in newly installed dental implants and their corresponding teeth in periodontally healthy individuals for six months, investigating the frequency and levels of putative periodontal species, as well as potential novel periodontal taxa of medical importance.

## Methodology

### Study population

This study included 19 healthy partially edentulous individuals assigned to receive single dental implants, who were recruited and treated from March to December 2016 at the Post-graduation Clinics, Universidade do Grande Rio. All participants signed an informed consent form after being informed about the study objectives. In addition to following STROBE guidelines, this study was conducted in full accordance with the revised Helsinki Declaration and approved by the Research Ethics Committee of the Universidade do Grande Rio (#1359026).

Prior to entering the study, all participants underwent a full periodontal examination. Inclusion criteria consisted of individuals >18 years of age with a correspondent tooth for the dental implant installed, <20% of sites with visible dental biofilm and <10% of sites with bleeding on probing, and no attachment loss due to periodontitis. Persisting bleeding after probing depth and clinical attachment level measurements was considered positive bleeding on probing. Visual inspection under relative isolation and proper illumination of the dental surface determined the presence of visible dental biofilm. Study exclusion criteria included: history of periodontitis; known diseases of the immune system (e.g., HIV-positive); diabetes; pregnancy or breastfeeding; necessity of chemoprophylaxis for dental care; antimicrobial use in the six months prior to the study; smoking; and periodontal treatment in the last year. Moreover, they should not be users of removable prosthesis or orthodontic appliances. Participants received no prophylactic antibiotics previously to implant placement surgery. To guarantee adequate oral hygiene control throughout the study, participants received personalized instructions on toothbrush (i.e., conventional, interproximal, etc.) and dental floss use at every visit.

### Sample size calculation

Sample size was calculated based on two standard deviations (5.27 and 5.41 counts x10^6^) of the mean counts of a bacterial species in samples from the same healthy individuals at two different timepoints.^24^ Detection of a $6\times 10^{6}$ difference required a minimum of 13 individuals with approximately 95% CI for the difference between means (D) D - 4.240 to D + 4.240. Sample size calculation was performed using a free software (WinPep, http://www.brixtonhealth.com/pepi4windows.html).

### Dental implant installation

Titanium alloy implants, manufactured according to ASTM standard F136, were installed in the posterior maxilla and mandibula using a one-stage open-flap technique, without surgical guide, with torques ranging from 35 to 60 N. All procedures were late installations with no graft addition as all surgical sites presented adequate keratinized mucosa width. Torques were measured using a manual torquemeter available on the surgical kit (Neodent^®^, Curitiba, PR, Brazil). A healing abutment was applied immediately after implant placement (SlimFit, Neodent^®^), followed by suture using Mononylon 5-0 (Mononylon^®^, J&J, São José dos Campos, SP, Brazil). All installed implants had an external hexagon connection (Neodent^®^), which are made of type 2 titanium alloy and have a surface treated by abrasive blasting followed by acid etching. Each participant received only one implant. We instructed all participants to use chlorhexidine at 0.12% twice a day for 7 days after the procedure and prescribed only analgesics. On day 7, we installed provisional crowns and checked occlusion by asking patients to bite the occlusion tape in all directions. Occlusion adjustments were made with a diamond bur at high rotation when necessary. The provisional crowns were kept until final evaluation. All provisional crowns were manufactured in acrylic resin by the same prosthetic laboratory using an addition silicone impression mold. The screw-retained prosthesis was obtained on a regular platform. We provided all participants with detailed and personalized hygiene instructions regarding dental brush, dental floss, and interdental brush, and made reinforcements at every visit when necessary.

### Microbiologic analysis

Subgingival biofilm samples were collected by the same trained examiner at 7, 30, 120, and 180 days after implant installation^5 , 14 , 25^ from all sites around the implant and its homologous tooth, when present, or its antagonist, which should not present bleeding on probing, in relative isolation using titanium sterile Gracey curettes (Hu-Friedy; Chicago, IL, USA) after cleaning the area of interest with sterile gauze. Samples were always collected first from the implant and then the tooth. Two pooled biofilm samples, one from each site, were obtained per patient. Sampling was performed before removal of the healing abutment on day 7, and with the provisional crowns in place at the remaining timepoints. The sampled pools were placed in individual microtubes containing 150 μL of tris-EDTA buffer (TE, 10 mM Tris HCl, 1 mM EDTA, pH 7.6), which were then filled with 150 μL of 0.5 M NaOH.

Microbiota composition was determined using checkerboard DNA-DNA hybridization.^26^ Briefly, the samples were lysed and fixed onto individual lanes on a nylon membrane (Molecular Dynamics GE Healthcare LifeSciences, Piscataway, NJ, USA) using a specific apparatus (Minislot 30, Immunetics, Cambridge, MA, USA), and hybridized against whole genomic digoxigenin-labeled probes by a hybridization apparatus (Miniblotter 45, Immunetics). DNA from *Aggregatibacter actinomycetemcomitans* ( *Aa* ) serotypes a, b and c, and *Cutibacterium acnes* I and II were pooled into two separate probes. Probes for other closely related species were pooled in equal concentrations, including *Actinomyces* spp., *Campylobacter* spp., *Capnocytophaga* spp., Enterics spp., *Eubacterium* spp., *Fusobacterium nucleatum* , *Gemella* spp., *Lactobacillus* spp., *Neisseria* spp., Milleri streptococci, Mitis streptococci, Mutans streptococci, *Prevotella* spp., and *Serratia* spp. ( Supplemental Table 1S ). We analyzed 85 species in total. Bound probes were detected by a digoxigenin phosphatase-conjugated antibody (Roche Applied Science, São Paulo, SP, Brazil) and fluorescence (AttoPhos®, Promega Corporation, Madison, WI, USA). Membrane signals captured and analyzed by an imaging system (Storm TM 860 and ImageQuant^®^ version 5.2, Molecular Dynamics GE Healthcare LifeSciences) were compared to the standards for the tested species, present on the same membrane at 100,000 and 1,000,000 bacterial cell counts. Signals were classified as: 0, not detected; 1, <100,000 cells; 2, approximately 100,000; 3, 100,000 to 1,000,000; 4, approximately 1,000,000; and 5, >1,000,000 cells. Assay sensitivity was adjusted to enable the detection of 10,000 cells of a given species by fixing each DNA probe concentration. This procedure was performed to provide the same detection sensitivity for each species. Failure to detect a signal was recorded as zero, although counts in the 1 to 1,000 ranges may have been present.

The microbiological analysis was performed by a blinded examiner.

### Data analysis

Statistical analyses were performed using a statistical software package (IBM SPSS Statistics Version 19, IBM, Armonk, USA). Mean levels (cell counts in a sample) and detection frequency of each target species were calculated for implants and teeth at days 7, 30, 120, and 180. Significant differences between teeth and implants were estimated by Mann-Whitney test, whereas changes in the microbiota over time were analyzed by Friedman test. Associations between implants or teeth and the levels of the species, which differed significantly between groups at the observation timepoints, were evaluated by linear regression analysis using the stepwise method. Significance level was set at 5%. Each group received a code to avoid bias during statistical analysis.

## Results

**Figure 1:**
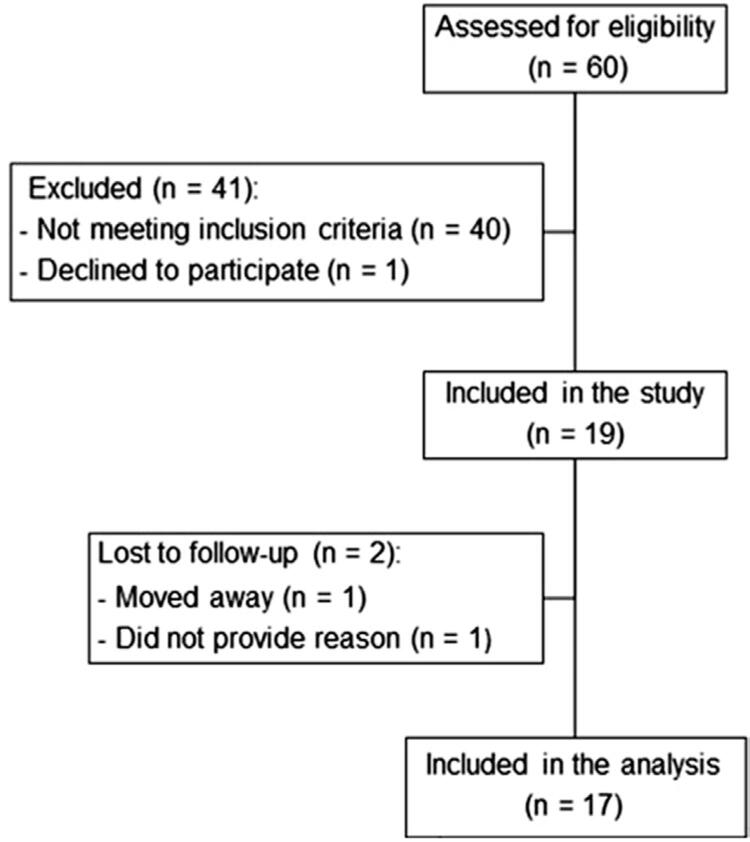
Flowchart of participant inclusion

A total of 19 eligible individuals, ten women (45 ± 10.8 years) and nine men (49 ± 15.5 years), participated in this study. During follow-up, two male patients dropped out (one moved away and another gave no explanation), thus, the current analysis considered only the individuals who took part in all periods of observation (n = 17), as illustrated in Figure 1 . We present the list of the dental implant location and the respective control tooth in a supplemental file ( Supplemental Table 2S ).

**Figure 2:**
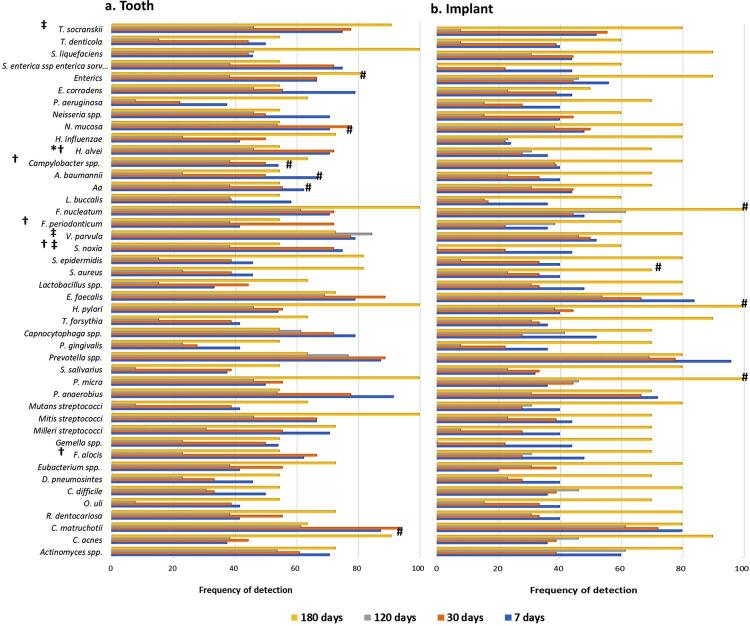
Detection frequency of the studied species in teeth and implant samples. (a) tooth samples; (b) implant samples; species: *S. enterica* ssp. *enterica sorv* , *Salmonella enterica* subsp. *enterica* serovar Typhi; *Significant difference between teeth and implants at 7 days, p <0.05, Mann-Whitney test; †Significant difference between teeth and implants at 30 days; ‡Significant difference between teeth and implants at 120 days; #Significant difference within group over time, Friedman test.

Figure 2 summarizes the detection frequency of the target species. Data from 07 and 30 days show that *Hafnia alvei* was detected in significantly higher frequency in teeth (70.8% and 72.2%, respectively) compared with implants (36% and 27.8%, respectively) (Mann-Whitney test; p<0.05). At 30 and 120 days, other species such as *Capnocytophaga* spp., *F. nucleatum, Filifactor alocis* , *Selenomonas noxia* , *Treponema socranskii* and *V. parvula* were also significantly more frequently detected in teeth compared with implants. We found no differences between implants and teeth at 180 days (p>0.05). Analysis over time showed that detection frequency increased significantly for *F. nucleatum, Helicobacter pylori, P. micra,* and *Staphylococcus aureus* in implant samples (Friedman test; p<0.05).

**Figure 3:**
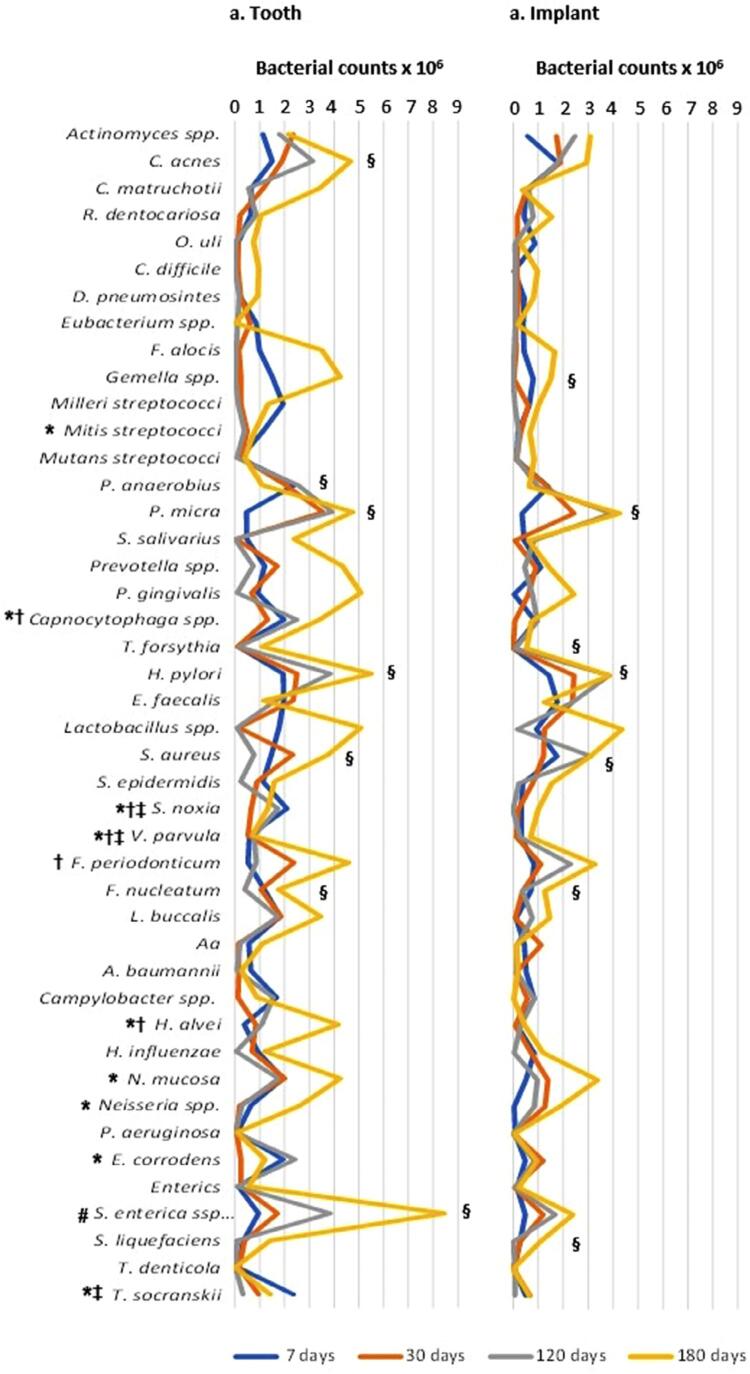
Levels of the studied species in teeth and implant samples. (a) tooth samples; (b) implant samples; species: *S. enteric* a ssp., *Salmonella enterica* subsp. *enterica* serovar Typhi; *Significant difference between teeth and implants at 7 days, p <0.05, Mann-Whitney test; †Significant difference between teeth and implants at 30 days; ‡Significant difference between teeth and implants at 120 days; #Significant difference between teeth and implants at 180 days; §Significant difference within group over time, Friedman test.

Figure 3 shows that some studied oral species presented levels significantly higher (p<0.05) in teeth compared to implants at day 07 ( *Capnocytophaga* spp., *E. corrodens, H. alvei,* Milleri streptococci, *N. mucosa, Neisseria* spp. *, S. noxia, T. socranskii* , and *V. parvula* ), day 30 ( *V. parvula* , *Capnocytophaga* spp., *Fusobacterium periodonticum* , *S. noxia,* and *H. alvei* ), day 120 ( *S. noxia, T. socranskii,* and *V. parvula* ), and day 180 ( *Salmonella enterica* subsp *. enterica* serovar Typhi). We found a significant increase in the levels of some species over time in teeth and implant samples ( *F. nucleatum* , *H. pylori, P. micra* , and *S. aureus).* Conversely *,* other species showed a level increase only in teeth ( *C. acnes* and *Salmonella enterica* subsp *. enterica* serovar Typhi) or in implants ( *Gemella* spp., *Serratia liquefaciens* and *Tannerella forsythia* ). Only one studied species decreased significantly over time in teeth samples ( *Peptostreptococcus anaerobius* ). We present the levels of the detected species grouped according to their phylum in a supplemental file ( Supplemental Figure 1S ; Supplemental Table 3S ).

Final linear regression models for each observation period showed that dental implants had significant negative correlations with the levels of *S. noxia* and *V. parvula* after 7 days ( Table 1 ). Only *S. noxia* remained in the final model 1, showing a standardized β coefficient (β) of -0.331 (p=0.02). In model 2, *S. noxia* (β=-0.288 ; p= 0.035 ) and *V. parvula* (β=-0.288; p=0.035) presented significant negative correlations with implants. In the 30-day analysis, only *S. noxia* showed a significant negative correlation with implants (β=-0.472; p=0.004). In model 2, however, *S. noxia* (β=-0.401 ; p= 0.009 ) and *V. parvula* (β= -0.354 ; p= 0.020) showed significant negative correlations with dental implants. At 120 days only *V. parvula* remained in the model, presenting a significant negative correlation with dental implants (β=-0.500; p=0.009). On day 180, only *S. enterica* subsp *. enterica* serovar Typhi had a significant negative correlation with dental implants (β=-0.639; p=0.001). LRA indicates that these species were less likely to be found in implant samples compared with teeth samples. As shown in these adjusted models, fewer species stood out at the observation periods, differently from the direct statistical comparison, in which a greater number of species presented significant differences.

**Table 1:** Linear regression analysis of the association between level of studied species and dental implant on the 6-month evaluation period

Predictor species included in the final model	Standardized β coefficient	P value	Adjusted R^2^
7 days*			
Model 1			
*S. noxia*	-0.331	0.020	0.091
Model 2			
*S. noxia*	-0.348	0.012	0.157
*V. parvula*	-0.288	0.035	
30 days†			
Model 1			
*S. noxia*	-0.472	0.004	0.199
Model 2			
*S. noxia*	-0.401	0.009	0.303
*V. parvula*	-0.354	0.020	
120 days‡			
*V. parvula*	-0.500	0.009	0.218
180 days§			
*S. enterica* subsp. *enterica* serovar Typhi	-0.639	0.001	0.380

*Species included in the step 1 for the analysis of day 7: *Capnocytophaga* spp., *E. corrodens* , *Milleri streptococci* , *N. mucosa* , *S. noxia* , *T. socranskii* , *V. parvula* , *H. alvei* , and *Neisseria* spp.; † Species included in the step 1 for the analysis of day 30: *C. sputigena* , *F. periodonticum* , *S. noxia* , *V. parvula* , and *H. alvei* . ‡Species included in the step 1 for the analysis of day 120: *S. noxia* , *T. socranskii* , and *V. parvula* ; §Species included in the step 1 for the analysis of day 180: *S. enterica* subsp. *enterica* serovar Typhi.

## Discussion

Our study evaluated the kinetics of subgingival microbiota colonization, analyzing classical bacterial complexes and novel periodontal pathogens established over time around newly installed implants in periodontally healthy individuals compared to their corresponding teeth. The levels of some species differed significantly between tooth and implant at one week after implant installation, whereas only *Salmonella enterica* subsp *. enterica* serovar Typhi showed higher levels in teeth compared to implants at six months. These results indicate that peri-implant sites promptly acquire a similar microbiota to the one found in the remaining teeth. Interestingly, the levels of these species leveled up over time between teeth and implants, corroborating data from other studies.^5 , 16 , 27^ After only one week in place, submucosal sites are already colonized by a diverse microbiota comparable to the one found in the subgingival healthy site, in line with other findings.^16^

According to the literature, any source or reservoir of potential pathogens (e.g., periodontal pockets) should be eradicated before dental implant installation to avoid microbiota dissemination to other areas,^3 , 7 , 21 , 28 , 29^ indicating that sites with dysbiotic subgingival biofilm might work as a reservoir for submucosal colonization. A previous study noted that implants with bacterial levels above 10,000 cells for the species *T. forsythia, Treponema denticola, Capnocytophaga rectus, T. socranskii, Porphyromonas gingivalis, S. aureus, Campylobacter gracilis,* and *Prevotella intermedia* had a significantly higher risk to present peri-implantitis with an odds ratio ranging from 3.1 to 4.6.^30^ Most of these species were detected above 10,000 cells in the present research, suggesting a potential risk of peri-implant disease onset even in periodontally healthy individuals since they can become periodontal/peri-implant diseased patients as they age. Moreover, current findings pointed out that periodontal pathogenic species show significant increase in levels over time, including *F. nucleatum* and *T. forsythia,* members of the orange and red complexes, respectively.^31^ Interestingly, colonization occurred even in patients who kept good dental/implant hygiene throughout the study period.

Our findings show that some species not usually associated with the subgingival site, such as *Filifactor alocis* , *H. pylori,* and *S. aureus* , are common in teeth and dental implants. Regarding periodontal disease, the implication of *F. alocis* in periodontal disease severity is well-described in clinical studies.^32 , 33^ Moreover, previous research has pointed out a potential role in periodontal disease pathogenesis for some species detected in the subgingival biofilm that are not usually associated with periodontal diseases, such as *Acinetobacter baumannii* and *Pseudomonas aeruginosa* .^34 , 35^ However, few studies explore the role of these bacteria and other “non-periodontal” species in peri-implant health or peri-implantitis.^30 , 36 , 37^ Relatively high levels of *Haemophilus influenzae, H. pylori,* and *S. aureus* may be detected in healthy dental implants or those presenting peri-implantitis.^30^ Another study detected *F. alocis* in 80%, *Staphylococcus epidermidis* in 29%, and *S. aureus* in 7.9% of the implant samples collected.^36^ Interestingly, some bacteria may be implant-specific, including *Propionibacteria, Paludibacter, Staphylococci, Filifactor* , and *Mogibacterium* .^38^ At the species level, another study found that *Rothia aeria, Streptococcus sanguinis, Streptococcus gordonii,* and *V. parvula* were the species most commonly associated with healthy submucosal sites *.* ^13^ Comparisons between the microbial profile of healthy and peri-implantitis sites show that their microbiota differs significantly, with peri-implantitis microbiota presenting greater diversity,^20^ including a wider list of statistically significant species related to peri-implant diseases.^23^ Our results demonstrate that the submucosal microenvironment is promptly colonized by a wide variety of species, even when considering periodontally healthy individuals, suggesting that a preventive program for individuals receiving dental implants should be established promptly.

The linear regression analysis showed that oral species *S. noxia* and *V. parvula* were essential for the colonization differences found from day 07 to 120. An early colonizer, *V. parvula* is considered a beneficial species and is clustered in the purple complex, as described by Socransky and Haffajee.^39^ *S. noxia* , in turn, does not cluster within any bacterial complex^39^ and seems to be more associated with healthy peri-implant than peri-implantitis sites.^4^ Another study also found a negative correlation between *S. noxia* and clinical parameters in peri-implant evaluation.^17^ Moreover, its detection increases over time in the peri-implant site of newly installed implants.^27^ Additional interesting data concerns the significant negative association between *S. enterica* subsp *. enterica* serovar Typhi and dental implants at 180 days. A previous research showed that the detection frequency and counts of this species tend to be higher in healthy sites compared to periodontitis sites, despite no significant differences detected.^35^ *S. enterica* subsp *. enterica* serovar Typhi is a gram-negative diarrheic enteropathogen with a virulence factor that can be found in other bacteria, such as *A. actinomycetemcomitans* , the cytolethal distending toxin.^40^ Most of the non-periodontal species analyzed here might be considered opportunistic pathogens and not oral cavity resident members. However, their clinical relevance and association with severe systemic infections justify further research on their interaction with periodontal and peri-implant health and disease.^35^

Following previous studies,^5 , 25^ we chose observation timepoints that would allow us to examine early submucosal colonization. At the 7-day timepoint, we were able to examine the immediate response of the host’s microbiota to the implant. By the 30-day timepoint, biofilm development is usually well underway, allowing us to investigate its composition and dynamics. Lastly, at the 120-day and 180-day timepoints we could glimpse the long-term effects of the implant on the microbiota. Biofilm is usually well-established by these later timepoints, and chronic interactions between the microbiota and the host have already occurred. Thus, these long-term observations can help us assess biofilm stability and evaluate any potential changes in microbial composition over time.^25^ But differently from those studies, the set of microorganisms analyzed here included species that are not commonly associated with periodontal/peri-implant diseases. As oral microbiological knowledge and technology advances, new relevant species are unveiled. Since our study included a great variety of species related to diseases/conditions in other parts of the human organism, the current findings can be used by future research to help target species that may present a higher risk for peri-implant diseases but not yet associated due to a lack of studies and evidence.

Despite the well-established causal relationship between the presence of pathogenic biofilm in peri-implant sites and the onset of peri-implant diseases,^1 , 12 , 20 , 23^ its colonization mechanisms require better understanding, including the interaction between the biomaterial and the biofilm and inflammatory response.^22^ Regarding implant material, research shows that zirconia and titanium are equally colonized by the bacteria present in the remaining teeth.^22^ Importantly, we used specific genomic probes to investigate only target species, which does not exclude the possibility of other bacteria being also associated with dental implants.

Most of the cited studies, such as the present research, included non-smokers, which represents a very restricted portion of the population with dental implants. Moreover, our study population included only individuals with periodontal health and good plaque or dental biofilm control. Thus, future studies should investigate not only the microorganism-biomaterial interaction but also how this is modulated by host risk factors. Another study limitation concerns the impossibility of blinding the examiner and the individuals to the intervention. To minimize bias, members of our group without knowledge of the sample codes performed all microbiological and statistical analyses.

Overall, our findings corroborate the literature,^5 , 25^ showing that submucosal sites are promptly colonized, even in individuals with good biofilm control. Hence, clinicians should provide proper care to the remaining teeth before considering dental implant installation and periodically monitor dental implants.

## Conclusion

Early submucosal microbiota is diverse and only a few species differ from teeth and implants in the same individual. Only seven days after installation, a rich microbiota can be found in the peri-implant site. After a 6-month evaluation, teeth and implants show similar prevalence and levels of the target species, including known and new periodontopathic species.
